# Isolated pulmonary regurgitation causes decreased right ventricular longitudinal function and compensatory increased septal pumping in a porcine model

**DOI:** 10.1111/apha.12904

**Published:** 2017-06-29

**Authors:** S. Kopic, S. S. Stephensen, E. Heiberg, H. Arheden, P. Bonhoeffer, M. Ersbøll, N. Vejlstrup, L. Søndergaard, M. Carlsson

**Affiliations:** ^1^ Department of Clinical Sciences Lund Clinical Physiology Skåne University Hospital Lund University Lund Sweden; ^2^ Department of Biomedical Engineering Faculty of Engineering Lund University Lund Sweden; ^3^ The Heart Centre Rigshospitalet Copenhagen University Hospital Copenhagen Denmark

**Keywords:** Cardiac magnetic resonance, mitral annular plane systolic excursion (MAPSE), Tetralogy of Fallot, tricuspid annular plane systolic excursion, ventricular function

## Abstract

**Aim:**

Longitudinal ventricular contraction is a parameter of cardiac performance with predictive power. Right ventricular (RV) longitudinal function is impaired in patients with free pulmonary regurgitation (PR) following corrective surgery for Tetralogy of Fallot (TOF). It remains unclear whether this is a consequence of the surgical repair, or whether it is inherent to PR. The aim of this study was to assess the relationship between longitudinal, lateral and septal pumping in a porcine model of isolated PR.

**Methods:**

Piglets were divided into a control (*n* = 8) group and a treatment (*n* = 12) group, which received a stent in the pulmonary valve orifice, inducing PR. After 2–3 months, animals were subjected to cardiac magnetic resonance imaging. A subset of animals (*n* = 6) then underwent percutaneous pulmonary valve replacement (PPVR) with follow‐up 1 month later. Longitudinal, lateral and septal contributions to stroke volume (SV) were quantified by measuring volumetric displacements from end‐diastole to end‐systole in the cardiac short axis and long axis.

**Results:**

PR resulted in a lower longitudinal contribution to RV stroke volume, compared to controls (60.0 ± 2.6% vs. 73.6 ± 3.8%; *P* = 0.012). Furthermore, a compensatory increase in septal contribution to RVSV was observed (11.0 ± 1.6% vs. −3.1 ± 1.5%; *P* < 0.0001). The left ventricle (LV) showed counter‐regulation with an increased longitudinal LVSV. Changes in RV longitudinal function were reversed by PPVR.

**Conclusion:**

These findings suggest that PR contributes to decreased RV longitudinal function in the absence of scarring from cardiac surgery. Measurement of longitudinal RVSV may aid risk stratification and timing for interventional correction of PR in TOF patients.

Ventricular function is a complex process, which is comprised of multiple sequential and parallel contractile events. In a simplified model, one can distinguish between a longitudinal – that is the movement of the atrioventricular plane (AVP) towards the apex of the heart – and a radial – that is the aggregate of the concentric contractions of both the lateral wall and the septum in the short‐axis plane – contribution to cardiac pumping. We have demonstrated that on average, 60% of left ventricular pumping and 80% of right ventricular pumping are attributable to longitudinal contraction by means of AVP displacement in the healthy human heart.^1^ These differences reflect an adaptation to the divergent ventricular and atrial filling processes and haemodynamic characteristics of the systemic and pulmonary circulation.^1^

It has become apparent that the analysis of the longitudinal contractile function is of clinical relevance in the assessment and prediction of cardiac pathologies, such as myocardial infarction, dilated cardiomyopathy, congenital heart disease and cardiac arrhythmias.^2^, ^3^, ^4^, ^5^, ^6^, ^7^, ^8^, ^9^, ^10^ Changes in longitudinal pumping can be observed before overall cardiac performance is compromised.^10^ Recently, cardiac magnetic resonance imaging (CMR) has emerged as an accurate approach for assessment of longitudinal function and its predictive nature.^7^ Thus, the characterization of the relationship between longitudinal and radial contribution to stroke volume has not only improved our understanding of cardiac physiology, but has also become an important clinical tool for better risk stratification in cardiac patients.

The pathophysiological changes underlying cardiac dysfunction in patients with Tetralogy of Fallot (TOF; pulmonary stenosis, overriding aorta, ventricular septal defect, right ventricular hypertrophy) have been the subject of ongoing investigation, as the optimal timing of follow‐up surgeries in these patients remains to be clearly defined.^11^ In particular, the timing and method for pulmonary valve replacement in patients with free pulmonary regurgitation (PR) after surgical repair using transannular patch remains controversial, as it requires a balance between maintaining right ventricular (RV) function and size on the one hand, and limiting the patient's exposure to multiple interventions throughout lifetime on the other.^11^, ^12^

The development of tools to further characterize cardiac function with the aim to aid in the clinical management of TOF patients with PR is therefore of importance and has been explored in several studies. For example, investigators observed reduced right ventricular AVP movement in patients with PR following corrective surgery for TOF.^13^, ^14^ Riesenkampff *et al*. recently reported impaired right atrial filling, which is functionally linked to AVP displacement, in TOF patients.^14^ However, the changes in atrial function were not observed in non‐TOF patients with isolated PR, that is patients who did not undergo open surgery, suggesting that the pathological cardiac parameters observed in TOF patients could be a by‐product of corrective surgery, rather than being inherent to PR.^15^

To address this controversy, the aim of this study was to investigate the effects of isolated PR on longitudinal and radial contributions to ventricular pumping in a porcine model. A better understanding of the pathophysiological changes underlying PR may lead to improved risk stratification and may aid in the clinical management of TOF patients with regard to timing of pulmonary valve replacement.

## Results

### Calculation of pulmonary and tricuspid regurgitation

To quantify the degree of PR, phase‐contrast imaging was conducted over the pulmonary artery. The average regurgitant fraction over the pulmonary valve was 42.9 ± 2.7% in the PR group (Table 1). Tricuspid regurgitation (TR) was observed in seven of 12 pigs in the PR group. In these pigs, TR amounted to 28.1 ± 6.47% (spread between 9.3% and 62.4%).

**Table 1:** Summary comparing volumetric and functional parameters between control animals (Ctrl), animals with pulmonary regurgitation (PR) and animals after percutaneous pulmonary valve replacement (PPVR)

	Control	PR	PPVR	p Ctrl v PR	p PR v PPVR	p Ctrl v PPVR
*n*	8	12	6			
PR %	1.3% ± 0.4%	42.9% ± 2.7%	1.0% ± 0.3%	**<0.0001**	**<0.0001**	0.5540
Weight basline kg^a^	13.5 ± 0.5	13.9 ± 0.8	14.9 ± 0.7	0.6615	0.3858	0.1345
Weight imaging kg^a^	54.9 ± 3.7	48.8 ± 4.7	76.5 ± 5.9	0.3302	**0.0041**	**0.0133**
LV						
LVEDV mL	106 ± 7	92 ± 6	137 ± 12	0.1244	**0.0111**	0.0606
LVEDVi % of THV	21.6% ± 0.5%	14.7% ± 0.9%	18.1% ± 0.5%	**<0.0001**	**0.0037**	**0.0002**
LVESV mL	45 ± 4	40 ± 3	49 ± 4	0.3449	0.0797	0.4411
LVESVi % of THV	9.0% ± 0.4%	6.4% ± 0.4%	6.6% ± 0.4%	**0.0005**	0.7188	**0.0022**
LVSV mL	62 ± 3	52 ± 4	87 ± 9	0.0612	**0.0087**	**0.0363**
LVEF %	58.4% ± 1.7%	56.4% ± 1.4%	63.2% ± 2.1%	0.3736	**0.0253**	0.1124
Longitudinal %	70.5% ± 2.1%	89.6% ± 4.0%	72.3% ± 2.0%	**0.0006**	**0.0014**	0.5394
Radial %	23.3% ± 1.3%	11.7% ± 3.6%	21.0% ± 2.6%	**0.0097**	0.0555	0.4543
Lateral %	20.3% ± 1.5%	37.0% ± 3.9%	22.6% ± 3.1%	**0.0015**	**0.0111**	0.5200
Septal %	3.0% ± 1.8%	−25.4% ± 3.5%	−1.7% ± 2.8%	**<0.0001**	**0.0001**	0.2025
RV						
RVEDV mL	105 ± 8	200 ± 13	167 ± 14	**<0.0001**	0.1069	**0.0045**
RVEDVi % of THV	21.3% ± 0.3%	31.4% ± 0.5%	22.3% ± 1.2%	**<0.0001**	**0.0002**	0.4718
RVESV mL	40 ± 4	81 ± 6	63 ± 4	**<0.0001**	**0.0287**	**0.0026**
RVESVi % of THV	8.0% ± 0.4%	12.7% ± 0.6%	8.5% ± 0.7%	**<0.0001**	**0.0005**	0.5583
RVSV mL	66 ± 4	120 ± 9	104 ± 10	**0.0032**	**0.0007**	**0.0093**
RVEF %	62.9% ± 1.9%	59.4% ± 1.7%	62.0% ± 1.8%	0.1859	0.3171	0.7262
RVEF corr %		27.0% ± 1.8%				
Longitudinal %	73.6% ± 3.8%	60.0% ± 2.6%	67.0% ± 1.0%	**0.0117**	**0.0235**	0.1377
Radial %	31.9% ± 2.5%	38.0% ± 1.9%	31.6% ± 2.7%	0.0721	0.0744	0.9233
Lateral %	34.7% ± 2.0%	27.0% ± 1.8%	30.4% ± 2.5%	**0.0113**	0.2921	0.1993
Septal %	−2.8% ± 1.7%	11.0% ± 1.6%	1.2% ± 2.4%	**<0.0001**	**0.0076**	0.2119

LV, left ventricle; RV, right ventricle; EDV, end‐diastolic volume; ESV, end‐systolic volume; EF, ejection fraction; radial, septal + lateral; EF corr., net pulmonary forward flow/EDV; THV, total heart volume. Bold values indicate p<0.05.

a For PR group: *n* = 8.

### Ventricular volumes

**Figure 1:**
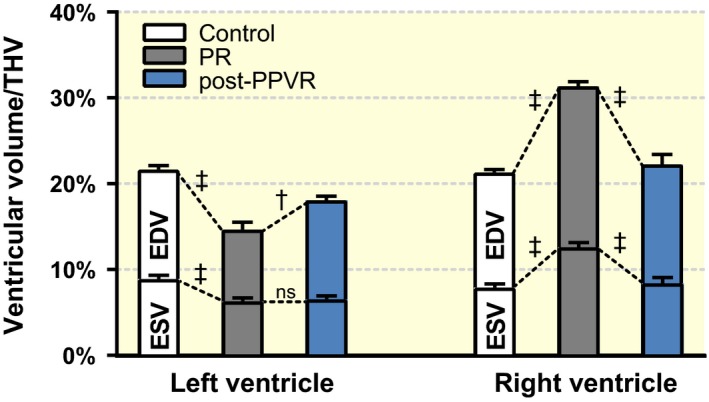
Ventricular volumes indexed as fractions of total heart volume (THV). Pulmonary regurgitation (PR; grey columns) results in a significant decrease in both left ventricular end‐diastolic (EDV) and end‐systolic volumes (ESV) expressed as fractions of THV, while the right ventricle increases its EDV and ESV as a result of volume overload. Percutaneous pulmonary valve replacement (PPVR; blue columns) restores ventricular volumes in the right ventricle. In the left ventricle, ESV remains unchanged, while EDV increases, albeit not to control levels (white columns). Columns (EDV and ESV) are not additive; hence, the difference between both columns represents the respective stroke volume normalized to THV. †*P* < 0.01; ‡*P* < 0.001, ns: non‐significant.

Ventricular volumes were indexed to total heart volume (THV). Both indexed left ventricular end‐diastolic volume (LVEDVi) and indexed left ventricular end‐systolic volume (LVESVi) showed a significant reduction in conditions of PR compared to control animals (LVEDVi: 14.7 ± 0.9% vs. 21.6 ± 0.5%; *P* < 0.0001; LVESVi: 6.4 ± 0.4% vs. 9.0 ± 0.4%; *P* < 0.001), while indexed right ventricular end‐diastolic volume (RVEDVi) and indexed right ventricular end‐systolic volume (RVESVi) were increased (RVEDVi: 31.4 ± 0.5% vs. 21.3 ± 0.3%; *P* < 0.0001; RVESVi: 12.7 ± 0.6% vs. 8.0 ± 0.4%; *P* < 0.0001; Fig. 1). Percutaneous pulmonary valve replacement (PPVR) normalized RVEDVi and RVESVi (PR vs. PPVR RVEDVi: 31.4 ± 0.5% vs. 22.3 ± 1.2%; *P* < 0.001; RVESVi: 12.7 ± 0.6% vs. 8.5 ± 0.7%; *P* < 0.001) and led to an increase in LVEDVi (PR vs. PPVR: 14.7 ± 0.9% vs. 18.1 ± 0.5%; *P* = 0.004). However, LVESVi remained unchanged (PR vs. PPVR: 6.4 ± 0.4% vs. 6.6 ± 0.4%; *P* = 0.719).

### Contributions to right ventricular stroke volume

**Figure 2:**
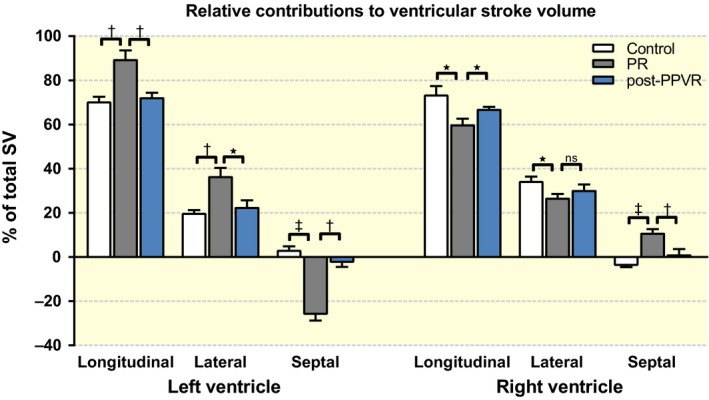
Individual contributions to ventricular stroke volume (SV). Pulmonary regurgitation (PR; grey columns) results in an increase in left ventricular longitudinal and lateral stroke volume compared to control animals (white columns). A marked negative septal contribution to left ventricular stroke volume can be observed in piglets with PR. In the right ventricle, PR causes reductions in both longitudinal and lateral pumping, while the septum significantly contributes to right ventricular stroke volume. Changes are mostly reversible 1 month after percutaneous pulmonary valve replacement (PPVR; blue columns). *P < 0.05; †*P* < 0.01; ‡*P* < 0.001, ns: non‐significant.

**Figure 3:**
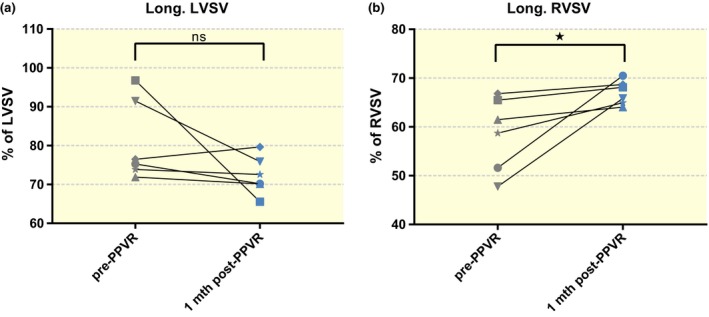
Before–after comparison of longitudinal pumping after percutaneous pulmonary valve replacement (PPVR). Individual pigs with pulmonary regurgitation (PR) who underwent PPVR showed a significant recovery of right ventricular longitudinal pumping 1 month after restoration of valve function (b). Changes in left ventricular longitudinal pumping were not significant (a). *P < 0.05; ns: non‐significant.

Pulmonary regurgitation resulted in a significantly lower contribution of longitudinal contraction to stroke volume (SV) in the RV compared to control animals (60.0 ± 2.6% vs. 73.6 ± 3.8%; *P* = 0.012; Fig. 2). Animals suffering from PR also demonstrated lower lateral contribution to right ventricular stroke volume (RVSV) (27.0 ± 1.8% vs. 34.7 ± 2.0%; *P* = 0.011), while septal contribution to RVSV was strongly increased in comparison with the physiological negative contribution to RVSV in healthy animals (11.0 ± 1.6% vs. −2.8 ± 1.7%; *P* < 0.0001; Fig. 2). Insertion of an artificial valve after PR restored longitudinal (PR: 60.0 ± 2.6%; PPVR: 67.0 ± 1.0%; *P* = 0.023) and septal (PR: 11.0 ± 1.6%; PPVR: 1.2 ± 2.4%; *P* = 0.008) RV function (Figs 2 and 3). Lateral contribution to RVSV did not demonstrate a significant recovery after valve insertion.

### Contributions to left ventricular stroke volume

In the left ventricle, animals with PR exhibited marked counter‐regulation with an increase in longitudinal left ventricular stroke volume (LVSV) (89.6 ± 4.0% vs. 70.5 ± 2.1%; *P* < 0.001), an increase in lateral LVSV (37.0 ± 3.9% vs. 20.3 ± 1.5%; *P* = 0.001) and a decreased septal LVSV (−25.4 ± 3.5% vs. 3.0 ± 1.8%; *P* < 0.0001; Fig. 2). PPVR reversed changes in longitudinal (PR: 89.6 ± 4.0%; PPVR: 72.3 ± 2.0%; *P* = 0.001), lateral (PR: 37.0 ± 3.9%; PPVR: 22.6 ± 3.1%; *P* = 0.011) and septal (PR: −25.4 ± 3.5%; PPVR: −1.7 ± 2.8%; *P* = 0.0001) LV contraction (Figs 2 and 3). Of note, changes in longitudinal and lateral contraction were not significant in a paired before–after analysis of the PPVR subgroup (Fig. 3). Functional and volumetric data are further summarized in Table 1.

### Correlation between corrected right ventricular ejection fraction and longitudinal left ventricular stroke volume

**Figure 4:**
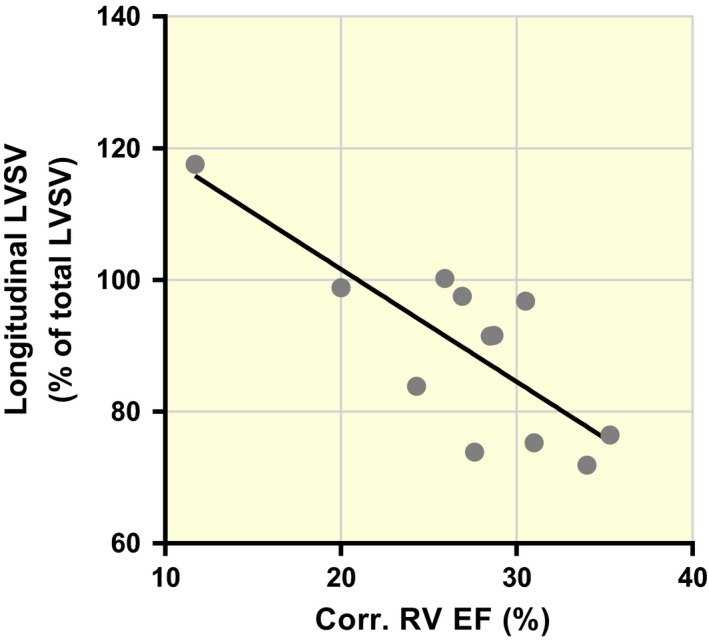
Correlation between corrected RV EF and longitudinal contribution to LVSV. Corrected RV EF is defined as the net pulmonary forward flow/RVEDV, that is RVSV minus pulmonary regurgitant volume and tricuspid regurgitant volume (if present); *R* ^2^=0.623; *P* = 0.002.

Animals with PR exhibited a correlation (*R* ^2^ = 0.623; *P* = 0.002) between the corrected RV ejection fraction (EF), that is net pulmonary forward flow/RVEDV and the longitudinal contribution to LVSV (Fig. 4).

### Summation of individual stroke volumes

As an internal quality control, the sum of calculated individual contributions to SV, that is longitudinal SV, lateral SV and septal SV, was compared to the SV obtained by delineation of the ventricles in end‐diastole (ED) and end‐systole (ES). For the LV, the summation of individual SVs amounted to 93.8 ± 2.2% in control, 101.0 ± 2.4% in PR and 93.3 ± 3.1 in PPVR animals respectively. In the RV, summation of individual SVs yielded a total of 104.9 ± 3.8% (control), 97.8 ± 1.7% (PR) and 98.6 ± 3.0% (PPVR) respectively.

### Invasive pressure measurements

Invasive pressure measurements were conducted in seven of eight animals in the control group (except for pulmonary capillary wedge pressure (PCWP) measurements: 6/8), eight of 12 pigs in the PR group and six of six animals in the PPVR group. Data are summarized in Table 2.

**Table 2:** Invasive pressure measurements

	Control	PR	PPVR	p Ctrl v PR	p PR v PPVR	p Ctrl v PPVR
*n*	7^a^	8	6			
RA sys	10 ± 2	13 ± 2	9 ± 2	0.2322	0.1166	0.7183
RA dia	6 ± 1	7 ± 2	4 ± 1	0.7257	0.2730	0.2699
RA mean	5 ± 1	6 ± 2	4 ± 1	0.3914	0.1415	0.4384
RV sys	35 ± 1	41 ± 4	53 ± 5	0.2712	0.0715	**0.0119**
RV dia	1 ± 1	2 ± 1	0 ± 0	0.4972	**0.0292**	0.1220
RV mean	10 ± 1	9 ± 1	9 ± 2	0.6748	0.9862	0.7060
PA sys	32 ± 2	25 ± 2	28 ± 2	**0.0316**	0.2874	0.1977
PA dia	10 ± 1	7 ± 2	10 ± 2	0.2800	0.4612	0.9930
PA mean	21 ± 2	15 ± 2	19 ± 1	**0.0196**	0.0855	0.3339
PCWP	10 ± 1	8 ± 1	9 ± 1	0.1042	0.4888	0.3643

RA, right atrium; RV, right ventricle; PA, pulmonary artery; PCWP, pulmonary capillary wedge pressure; sys, systolic; dia, diastolic; PPVR, percutaneous pulmonary valve replacement; Ctrl, control. Bold values indicate p<0.05.

a For PCWP measurements: 6; all measurements in mmHg.

### Correlation between longitudinal contribution to right ventricular stroke volume and pulmonary capillary wedge pressure

**Figure 5:**
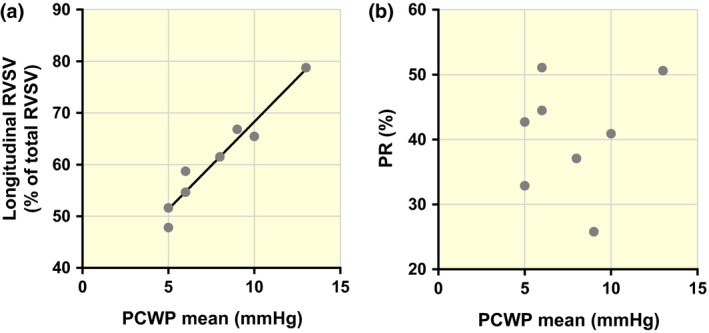
Correlations between mean PCWP and longitudinal contribution to RVSV (a) and PR (b). a: PCWP correlates with the degree of longitudinal contribution to RVSV in animals with PR (*R* ^2^=0.940; *P* < 0.001). b: PCWP does not correlate with the degree of PR (*R* ^2^=0.020; *P* = 0.738).

Animals in the PR group demonstrated a correlation between the longitudinal contribution to RVSV and mean PCWP (*R* ^2^ = 0.940; *P* < 0.001) (Fig. 5a). No correlation could be found between PCWP and the degree of pulmonary regurgitation (*R* ^2^ = 0.020; *P* = 0.738; Fig. 5b).

### Interobserver variability

The average interobserver variability for both ventricular volumes combined was 0.6 ± 5.1% and for AVP measurements 4.9 ± 13.7% (data presented as mean ± SD).

## Discussion

This study analyses the individual contributions of longitudinal, lateral and septal contraction to LVSV and RVSV in pigs suffering from isolated PR. The results suggest that decreased longitudinal function in the RV is a result of PR and corresponding RV volume overload and not only a consequence of cardiac surgery.

Severe PR is a common feature following corrective surgery for TOF and entails numerous long‐term complications, including exercise impairment, ventricular dysfunction, arrhythmias and sudden cardiac death.^16^, ^17^ As long‐term survival increases and increasing numbers of primarily operated children with TOF advance in age, strategies for the long‐term management of PR in this population are being developed. The challenge hereby rests in preserving RV function, while keeping the number of corrective interventions for PR in the course of the patient's life at a minimum. To this end, CMR cut‐off volumes for the RV are being proposed to determine the optimal time point of pulmonary valve replacement, although the exact volume threshold for intervention is still under debate.^18^ The use of cut‐off volumes as a factor supporting decision‐making, however, does not encompass a functional perspective of the RV. This problem is highlighted by recent longitudinal observations, demonstrating that RV dilation does not necessarily progress in TOF patients and reaches a stable plateau.^19^, ^20^

Recently, it has become evident that assessment of AVP displacement or longitudinal contraction, whether by CMR or echocardiography, serves as a potent predictor of adverse events in a range of cardiac pathologies including TOF.^3^, ^7^, ^9^ For example, it has been demonstrated that longitudinal LV dysfunction assessed by echocardiography is a predictor for severe arrhythmias and death in TOF patients.^3^ Despite the lack of prospective data, it is also apparent that TOF patients with free PR suffer from reduced longitudinal contribution to RVSV and exhibit pathological right atrial filling and a decreased right atrial reservoir function, as evidenced by recent insight by Riesenkampff *et al*.^13^, ^14^, ^15^ The authors argue that decreased atrial reservoir function observed in TOF patients is a by‐product of surgery, as evidenced by the lack of atrial abnormalities in non‐TOF patients with isolated PR.^14^, ^15^ The data presented in this manuscript, however, support the idea that isolated PR also results in reduced longitudinal RV pumping and is therefore independent of surgery or scarring. Atrial filling and longitudinal ventricular pumping are closely intertwined processes.^1^, ^2^, ^14^, ^21^, ^22^, ^23^ The displacement of the AVP during ventricular systole draws blood into the atria, similar to the piston aspirating liquid into a syringe. Given the interdependence between the reservoir function of the atria and the extent of displacement of the AVP in ventricular systole, the discrepancy between the observations of Riesenkampff *et al*. and this study remains to be elucidated. Alas Riesenkampff *et al*.^15^ did not assess parameters of longitudinal function in their non‐TOF patient population with PR, nor does this study directly address atrial function. Despite the apparent differences between the two studies, that is porcine model vs. patients, duration of PR, etc. (see *Limitations*), it is worth noting the following: the cohort with isolated PR (non‐TOF patients) in Riesenkampff *et al.'s* study did not have significantly enlarged RVEDV (indexed to body surface area), compared to control subjects, while the TOF patients with PR did.^15^ It thus remains to be clarified whether patients with close to normal RVEDV and isolated PR harbour a latent atrial or longitudinal pumping dysfunction that may be uncovered as the enlargement of the RV progresses.

The degree of reduction in longitudinal RVSV in this porcine model is consistent, albeit to a lesser extent, with our observations made in TOF patients with PR.^13^ While reduced RV global longitudinal strain has recently been described in an echocardiographical study in TOF patients with PR, it should be noted that no reduction in RV strain or tricuspid annular plane systolic excursion (TAPSE) has been noted in this animal model of free PR previously.^24^, ^25^, ^26^ While TAPSE is an adequate measure of AVP excursion, this study assessed the volumetric contribution of longitudinal pumping to overall stroke volume. Hence, TAPSE may be unaffected, while longitudinal pumping from a volumetric perspective may well display alterations. A similar rationale may be applied to strain measurements.

We have previously postulated that the reduction in RV longitudinal pumping may be an adaptive mechanism with the aim of more adequately matching right atrial (RA) inflow to net RVSV, that is SV minus regurgitant volume, as a preserved longitudinal function would result in overfilling of the atria and a concomitant pendulum volume.^13^ The observations made in this study further substantiate this hypothesis.

We have also observed a correlation between the degree of RV longitudinal pumping and PCWP (Fig. 5a). Although conclusions with regard to this effect remain speculative, we hypothesize that the mechanisms for this observation are an underfilling of the LV in this model of PR. The LV demonstrates signs of underfilling, as suggested by a low LVSV. Low PCWP is regarded as a proxy for left atrial pressure, and thus is directly coupled to LV filling. The fact that there was no correlation between the degree of PR and PCWP, but a very strong correlation between RV longitudinal pumping and PCWP, indicates that RV longitudinal pumping has added value as a functional parameter over the degree of valvular lesion.

To compare ventricular volumes between different experimental groups, we have indexed ventricular volumes as fractions of THV. As THV remains almost constant during the cardiac cycle and the four chambers of the heart are volumetrically coupled, THV can be used as a volumetric control accounting for different animal growth rates.^27^ We observed a marked increase in RVEDVi and RVESVi due to RV volume overload. Both parameters reversed to normal levels after restoration of valve function. Conversely, LVEDVi and LVESVi decreased during conditions of PR, most likely reflecting LV underfilling. This is further substantiated by the pronounced paradoxical spetal movement, resulting in a septal contribution to RVSV (Fig. 2). This observation is in line with previous findings in TOF patients.^13^ Paradoxical septal movement is a common sign of RV volume overload.^28^ It has been suggested that the diastolic bulging of the septum into the LV due to RV distension causes a decrease in LVEDV and therefore results in an adverse functional coupling of both ventricles as RV overload progresses.^29^ A reduction in LVEDV has been reported in TOF patients.^13^ Interestingly, paradoxical septal movement has also been observed in TOF patients without PR and RV overload. However, this abnormality is attributable to changes in excitation propagation, specifically a right bundle branch block.^30^ While paradoxical septal movement in TOF patients with PR is therefore most likely a combination of both RV overload and abnormal conductivity, we attribute the observed paradoxical septal movement in this model primarily to volume overload.

As septal contraction contributes to LVSV in the healthy heart, paradoxical septal movement will negatively affect LVSV. We have previously observed an increase in lateral contribution to LVSV in TOF patients to compensate for the loss of septal contribution to LVSV.^13^ In contrast to our previous findings, we here report a compensatory increase in longitudinal and lateral contribution to LVSV. This discrepancy may be attributable to a more acute adaptation to RV overload (2–3 months) compared to TOF patients, where adaptation evolves over several years, or may alternatively be unique to the porcine model (see *Limitations*). Ultimately, the underlying cause of this difference cannot be conclusively elucidated at this point; however, the correlation between the amount of right ventricular impairment and the degree to which the left ventricle increases its longitudinal contribution to SV may point towards a functional importance of this adaptive mechanism in this model where there is an acute onset of PR. A potential explanation for this observation may be found in the physiology of systolic left atrial filling, which is primarily driven by ventricular longitudinal pumping. With progressing right ventricular functional impairment, the left ventricle may support left atrial filling by increasing its longitudinal contribution to SV, with the overall aim of maintaining energetically favourable constant total heart volume.^27^ This hypothesis would also be in line with the observed indicators of impaired LV filling, such as low LVSV. Alternatively, the increase in longitudinal pumping may simply reflect a compensation for the loss of septal contribution to SV. This is supported by previous observations in patients with pulmonary arterial hypertension (PAH). These patients exhibit LV underfilling and an intact septal contribution to LVSV.^31^ Contrarily to our observation, LV longitudinal pumping in PAH is, however, reduced, suggesting that the increased longitudinal contribution to LVSV in PR may in part be a compensation for lost septal SV due to paradoxical septal movement.^31^ Despite our speculative understanding of the pathophysiological mechanisms underlying LV pumping adaptation to PR, it is worth stressing the fundamental observation that the LV has the capacity to adapt its pumping mechanics to RV impairment and isolated loss of LV function, that is septal pumping, and maintain effective SV in the process.

This study further demonstrates a general reversibility of the changes in cardiac pumping induced by PR and allows speculation about the nature of the adaptive mechanism. The observed changes may be a result of cardiac remodelling or alternatively a purely mechanical response to altered volume/pressure relationships. While this study cannot conclusively delineate between both mechanisms, it can be noted that 1 month of restored valve function, which in turn can be considered a short to medium time period for reversal of remodelling effects, almost entirely normalized cardiac pumping. Previous histological studies in this animal model further demonstrate that the muscle hypertrophy resulting from PR is still present 1 month after valve insertion.^32^ We therefore speculate that changes in longitudinal pumping are mostly mechanic, rather than structural adaptations; however, more acute measurements after restoration of valve function will be necessary to further elucidate this question.

### Study limitations

We observed TR in seven of 12 pigs with PR. It can be assumed that TR manifests in these cases secondarily to PR‐induced RV distension. Subgroup analysis between TR vs. non‐TR animals revealed a statistical difference in RVESVi in the PR group (TI: RVESVi = 11.7 ± 0.6%; non‐TI: 14.0 ± 0.6%; *P* = 0.026). We attribute this difference to the need for generation of a larger SV during conditions of TR, in order to maintain a constant net pulmonary forward SV. While other isolated effects of TR could not be identified, an influence on pumping mechanics cannot be conclusively excluded due to the small number of animals available for subgroup analysis.

The acute onset of free PR in the present study reflects the clinical situation when using a transannular patch in TOF repair, and the degree of PR in the animal model is generally comparable to the TOF patient population (e.g. 39 ± 3% in^13^ vs. 42.9 ± 2.7% in this study). However, in this animal model, the RV was prior to the intervention not pressure overloaded compared to pressure overload occurring in TOF cases. Also the limited duration (2–3 months) was much shorter than the decades often seen in humans with free PR. However, these few months represent a growth from piglet to adult pigs and, furthermore, caused a more pronounced increase in RV volume (factor 2.3 ± 0.3 compared to LVEDV) than typically occurring in patients (e.g. factor 1.7). This change may therefore evoke different adaptive mechanisms than in the clinical context, where PR and RV volume overload evolves gradually and persists over years.^33^

A further limitation of this study is that not all animals have been exposed to the same duration of PR (2 or 3 months). This is attributable to the fact that the original experimental protocol aimed at answering a different scientific question, namely the effectiveness of PPVR to rescue different degrees of RV dysfunction after induction of PR.^34^ In this analysis, the subgroups were pooled to increase sample size. Furthermore, it has been documented that the largest dynamic increase in parameters, such as RVEDV, occurs within the first month of PR, implying that functional differences between 2 and 3 months may not be as pronounced compared to earlier time points.^34^

While insertion of a stent is an effective way of inducing isolated PR an influence of the stent on AVP movement or other pumping mechanics cannot be excluded.

## Conclusions

This study underlines the value of characterizing the individual contributions to SV, as functional impairments, particularly in longitudinal pumping, are present before more global parameters, such as EF, are affected. While not offering prospective information, this study shows the value of longitudinal contractile function as a routine marker in the assessment of RV function, particularly in TOF patients. Measurement of longitudinal RVSV may support risk stratification and facilitate timing for interventional correction of PR. Furthermore, this study demonstrates that the observed changes are inherent to PR and therefore occur independently of previous corrective surgery.

## Materials and methods

### Animals

The design of the experimental study has been previously published.^24^, ^32^, ^34^ In short, piglets (cross‐breeds of Danish Landraces, Yorkshire and Duroc) were divided into control (*n* = 8) and treatment (*n* = 12) groups. At baseline, piglets were 8–9 weeks old. Animals in the treatment group received a bare metal stent in the pulmonary valve orifice via percutaneous right heart catheterization, inducing free PR, while animals in the control group underwent sham interventions. PR was maintained for 2 or 3 months, before re‐examination. A subset of animals in the treatment group (*n* = 6) underwent PPVR after 2–3 months of PR and was subjected to re‐examination 1 month after valve repair. The animal study was approved by the Animal Experiments Inspectorate (Danish Ministry of Justice, ref. no. 2005/561/1010).

### Interventions

Before intervention, animals received sedation with midazolam 0.5 mg/kg. Subsequently, anaesthesia was induced and maintained with intramuscular injection of a mixture of tiletamine, zolazepam, xylazine, ketamine and methadone. Animals breathed spontaneously (40% O_2_); however, intubation was performed to secure the airways. During intervention, amiodarone 5 mg/kg was administered intravenously to reduce occurrence of cardiac arrhythmias. Heparin was given as an anticoagulant (start dose 100 IU/kg, followed by 50 IU/kg/30 min maintenance). Percutaneous right heart catheterization was performed through the femoral vein. Following angiography (Omnipaque; GE Health Care, Piscataway, NJ, USA), a bare CP^®^ stent (NuMED, Hopkinton, NY, USA) mounted on a 22‐mm BIB^®^ dilation catheter (NuMED) was advanced to the pulmonary valvular annulus inducing free PR. Invasive pressure measurements (RA, RV, pulmonary artery (PA) and PCWP) were conducted during catheterization using a fluid‐filled transducer. For animals in the PPVR group, a Medtronic Melody^®^ valve (Medtronic, Minneapolis, MN, USA) was placed inside the CP^®^ stent following 2–3 months of free PR. Adequate valve function was confirmed with pulmonary angiography and echocardiography.

### Cardiac magnetic resonance imaging

Cardiac magnetic resonance imaging was conducted on a 1.5T scanner (Siemens MAGNETOM^®^ Vision, Erlangen, Germany), equipped with a standard phased array chest coil. Imaging was performed after two (*n* = 7) and three (*n* = 5) months of PR. Control animals (*n* = 8) were imaged at corresponding time points. For animals in the PPVR group (2 months of PR: *n* = 4; 3 months of PR: *n* = 2), imaging was repeated 1 month after valve insertion. Short‐ and long‐axis images were acquired with a cinematographic gradient echo pulse sequence (temporal resolution: 30 ms; slice thickness: 6 mm; matrix size: 256 × 256). To calculate pulmonary regurgitation volume, velocity‐encoded phase‐contrast cine images were acquired over the main pulmonary artery (temporal resolution: 24 ms; slice thickness: 6 mm; matrix size: 256 × 256, VENC 250 cm/s).

### Image analysis

**Figure 6:**
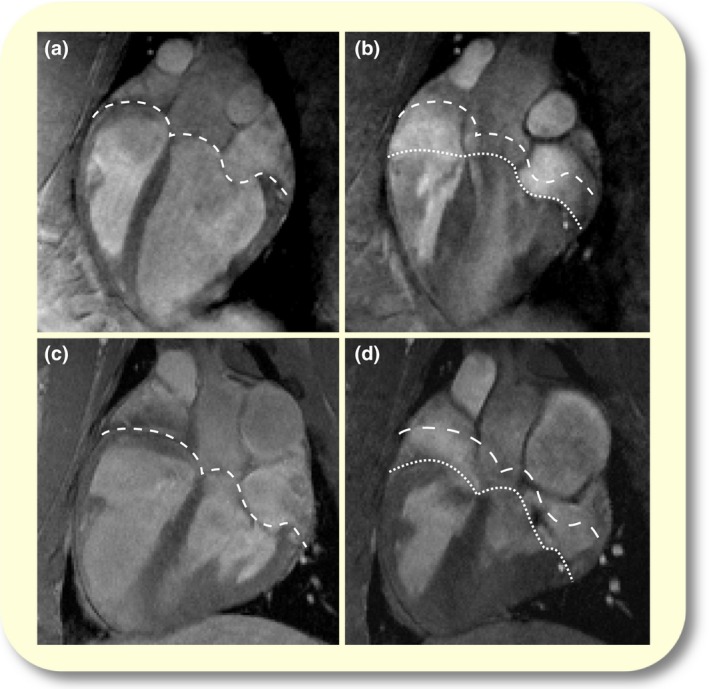
Long‐axis view of the heart. Panels (a) and (b) show the heart of a control animal in end‐diastole (a) and end‐systole (b) respectively. Dashed line represents the AVP in end‐diastole. Dotted line represents the AVP in end‐systole. Panels c and d depict the heart of an animal suffering from PR (3 months) at corresponding imaging time points. Note the increased RV volume and the decreased RV AVP movement with a compensatory increase in LV AVP movement.

Image analysis was performed using the image analysis software Segment (v2.0, R5100; Medviso, www.medviso.com).^35^ LV and RV volumes were calculated by manual delineation of endocardial borders in all short‐axis slices in end‐diastole (ED) and end‐systole (ES). To allow for intergroup comparison, ventricular volumes were indexed to the respective THV and expressed as percentages thereof. The individual contributions to ventricular SV, that is the volumes displaced by longitudinal, lateral and septal contraction (lateral and septal contributions can further be consolidated under the term radial contraction), were calculated, as described previously.^1^, ^2^ In brief, for calculation of longitudinal SV, the position of the AVP was determined in ED and ES by on average seven measurement points in long‐axis views. Representative examples and corresponding AVP displacement are shown in Figure 6. The displacement of the AVP from ED to ES was multiplied by the average of the three largest areas enclosed by the ventricular epicardial short‐axis contours, yielding the longitudinal contribution to SV. For the rationale underlying this method, please refer to the studies of Carlsson *et al*. 1, 2 By tracing the epicardial contours and defining the ventricular septum, the volumes generated by lateral and septal displacement between ED and ES were calculated and their corresponding contribution to SV was determined.^1^, ^2^, ^13^ PR was calculated as the percentage of retrograde blood flow of total forward flow in the pulmonary artery. Presence of TR was determined by visual signs in the MR images, such as visible flow artefacts over the tricuspid valve during ventricular systole, and quantified using the discrepancy between pulmonary forward flow volumes and delineated ventricular stroke volumes.

Interobserver variability was calculated by blinded comparison of ventricular volumes and AVP measurements in ten data sets between the authors S.K. and S.S.S.

### Statistical analysis

Data are presented as means ± standard error of the mean (SEM). Welch's unpaired *t*‐test was used to test for differences in means between two populations. A paired *t*‐test was used to compare individual differences before and after PPVR in the PPVR subset. Interobserver variability was calculated as the mean bias ± standard deviation. Statistical significance was assumed at results with a *P*‐value <0.05.

## Conflict of interest

Lars Sondergaard is a consultant for and has received unrestricted research grants from Medtronic. Philipp Bonhoeffer is a consultant to Medtronic and NuMED and has received honoraria and royalties for the device described. Einar Heiberg is founder and owner of Medviso AB producing the Segment software for medical image analysis. NuMED, Hopkinton, NY, USA, and Medtronic Minneapolis/St Paul, MN, USA, are acknowledged for financial support to the study.

This work was supported by the Swedish Research Council, Swedish Heart Lung Foundation, the Swedish Medical Association, Lund University, the Region of Skåne and the Danish Heart Foundation. NuMED, Hopkinton, NY, USA, and Medtronic Minneapolis/St Paul, MN, USA, are acknowledged for financial support to the study.

## References

[1] Carlsson M , Ugander M , Heiberg E , Arheden H : The quantitative relationship between longitudinal and radial function in left, right, and total heart pumping in humans. Am J Physiol Heart Circ Physiol 293: H636–H644, 2007. 17307988 10.1152/ajpheart.01376.2006

[2] Carlsson M , Ugander M , Mosen H , Buhre T , Arheden H : Atrioventricular plane displacement is the major contributor to left ventricular pumping in healthy adults, athletes, and patients with dilated cardiomyopathy. Am J Physiol Heart Circ Physiol 292: H1452–H1459, 2007. 17098822 10.1152/ajpheart.01148.2006

[3] Diller GP , Kempny A , Liodakis E , Alonso‐Gonzalez R , Inuzuka R , Uebing A , Orwat S , Dimopoulos K , Swan L , Li W , Gatzoulis MA , Baumgartner H : Left ventricular longitudinal function predicts life‐threatening ventricular arrhythmia and death in adults with repaired tetralogy of fallot. Circulation 125: 2440–2446, 2012. 22496160 10.1161/CIRCULATIONAHA.111.086983

[4] Henein MY , Gibson DG : Long axis function in disease. Heart 81: 229–231, 1999. 10026340 10.1136/hrt.81.3.229 PMC1728969

[5] Hu K , Liu D , Herrmann S , Niemann M , Gaudron PD , Voelker W , Ertl G , Bijnens B , Weidemann F : Clinical implication of mitral annular plane systolic excursion for patients with cardiovascular disease. Eur Heart J Cardiovasc Imaging 14: 205–212, 2013. 23161791 10.1093/ehjci/jes240

[6] Kalam K , Otahal P , Marwick TH : Prognostic implications of global LV dysfunction: a systematic review and meta‐analysis of global longitudinal strain and ejection fraction. Heart 100: 1673–1680, 2014. 24860005 10.1136/heartjnl-2014-305538

[7] Rangarajan V , Chacko SJ , Romano S , Jue J , Jariwala N , Chung J , Farzaneh‐Far A : Left ventricular long axis function assessed during cine‐cardiovascular magnetic resonance is an independent predictor of adverse cardiac events. J Cardiovasc Magn Reson 18: 35, 2016. 27266262 10.1186/s12968-016-0257-y PMC4897936

[8] Rydberg E , Arlbrandt M , Gudmundsson P , Erhardt L , Willenheimer R : Left atrioventricular plane displacement predicts cardiac mortality in patients with chronic atrial fibrillation. Int J Cardiol 91: 1–7, 2003. 12957723 10.1016/s0167-5273(02)00578-8

[9] Willenheimer R , Cline C , Erhardt L , Israelsson B : Left ventricular atrioventricular plane displacement: an echocardiographic technique for rapid assessment of prognosis in heart failure. Heart 78: 230–236, 1997. 9391283 10.1136/hrt.78.3.230 PMC484923

[10] Ersboll M , Valeur N , Andersen MJ , Mogensen UM , Vinther M , Svendsen JH , Moller JE , Kisslo J , Velazquez EJ , Hassager C , Sogaard P , Kober L : Early echocardiographic deformation analysis for the prediction of sudden cardiac death and life‐threatening arrhythmias after myocardial infarction. JACC Cardiovasc Imaging 6: 851–860, 2013. 23850252 10.1016/j.jcmg.2013.05.009

[11] Burchill LJ , Wald RM , Harris L , Colman JM , Silversides CK : Pulmonary valve replacement in adults with repaired tetralogy of Fallot. Semin Thorac Cardiovasc Surg Pediatr Card Surg Annu 14: 92–97, 2011. 21444054 10.1053/j.pcsu.2011.01.016

[12] Kim YY , Ruckdeschel E : Approach to residual pulmonary valve dysfunction in adults with repaired tetralogy of Fallot. Heart 102: 1520–1526, 2016. 27329296 10.1136/heartjnl-2015-309067

[13] Stephensen S , Steding‐Ehrenborg K , Munkhammar P , Heiberg E , Arheden H , Carlsson M : The relationship between longitudinal, lateral, and septal contribution to stroke volume in patients with pulmonary regurgitation and healthy volunteers. Am J Physiol Heart Circ Physiol 306: H895–H903, 2014. 24441546 10.1152/ajpheart.00483.2013

[14] Riesenkampff E , Mengelkamp L , Mueller M , Kropf S , Abdul‐Khaliq H , Sarikouch S , Beerbaum P , Hetzer R , Steendijk P , Berger F , Kuehne T : Integrated analysis of atrioventricular interactions in tetralogy of Fallot. Am J Physiol Heart Circ Physiol 299: H364–H371, 2010. 20495149 10.1152/ajpheart.00264.2010 PMC2930385

[15] Riesenkampff E , Al‐Wakeel N , Kropf S , Stamm C , Alexi‐Meskishvili V , Berger F , Kuehne T : Surgery impacts right atrial function in tetralogy of Fallot. J Thorac Cardiovasc Surg 147: 1306–1311, 2014. 23896323 10.1016/j.jtcvs.2013.06.020

[16] Gatzoulis MA , Balaji S , Webber SA , Siu SC , Hokanson JS , Poile C , Rosenthal M , Nakazawa M , Moller JH , Gillette PC , Webb GD , Redington AN : Risk factors for arrhythmia and sudden cardiac death late after repair of tetralogy of Fallot: a multicentre study. Lancet 356: 975–981, 2000. 11041398 10.1016/S0140-6736(00)02714-8

[17] Knauth AL , Gauvreau K , Powell AJ , Landzberg MJ , Walsh EP , Lock JE , del Nido PJ , Geva T : Ventricular size and function assessed by cardiac MRI predict major adverse clinical outcomes late after tetralogy of Fallot repair. Heart 94: 211–216, 2008. 17135219 10.1136/hrt.2006.104745

[18] Lee C , Kim YM , Lee CH , Kwak JG , Park CS , Song JY , Shim WS , Choi EY , Lee SY , Baek JS : Outcomes of pulmonary valve replacement in 170 patients with chronic pulmonary regurgitation after relief of right ventricular outflow tract obstruction: implications for optimal timing of pulmonary valve replacement. J Am Coll Cardiol 60: 1005–1014, 2012. 22921969 10.1016/j.jacc.2012.03.077

[19] Greutmann M : Tetralogy of Fallot, pulmonary valve replacement, and right ventricular volumes: are we chasing the right target? Eur Heart J 37: 836–839, 2016. 26685132 10.1093/eurheartj/ehv634

[20] Rutz T , Ghandour F , Meierhofer C , Naumann S , Martinoff S , Lange R , Ewert P , Stern HC , Fratz S : Evolution of right ventricular size over time after tetralogy of Fallot repair: a longitudinal cardiac magnetic resonance study. Eur Heart J Cardiovasc Imaging 18: 364–370, 2017. 28363200 10.1093/ehjci/jew273

[21] Barbier P , Solomon SB , Schiller NB , Glantz SA : Left atrial relaxation and left ventricular systolic function determine left atrial reservoir function. Circulation 100: 427–436, 1999. 10421605 10.1161/01.cir.100.4.427

[22] Fujii K , Ozaki M , Yamagishi T , Ishine K , Furutani Y , Nagano H , Yamamoto K , Saiki A , Matsuzaki M : Effect of left ventricular contractile performance on passive left atrial filling–clinical study using radionuclide angiography. Clin Cardiol 17: 258–262, 1994. 8004840 10.1002/clc.4960170508

[23] Steding‐Ehrenborg K , Carlsson M , Stephensen S , Arheden H : Atrial aspiration from pulmonary and caval veins is caused by ventricular contraction and secures 70% of the total stroke volume independent of resting heart rate and heart size. Clin Physiol Funct Imaging 33: 233–240, 2013. 23522018 10.1111/cpf.12020

[24] Kjaergaard J , Iversen KK , Vejlstrup NG , Smith J , Bonhoeffer P , Sondergaard L , Hassager C : Effects of chronic severe pulmonary regurgitation and percutaneous valve repair on right ventricular geometry and contractility assessed by tissue Doppler echocardiography. Echocardiography 27: 854–863, 2010. 20546000 10.1111/j.1540-8175.2010.01153.x

[25] Kjaergaard J , Iversen KK , Vejlstrup NG , Smith J , Bonhoeffer P , Sondergaard L , Hassager C : Impacts of acute severe pulmonary regurgitation on right ventricular geometry and contractility assessed by tissue‐Doppler echocardiography. Eur J Echocardiogr 11: 19–26, 2010. 19812060 10.1093/ejechocard/jep149

[26] Li Y , Xie M , Wang X , Lu Q , Zhang L , Ren P : Impaired right and left ventricular function in asymptomatic children with repaired tetralogy of Fallot by two‐dimensional speckle tracking echocardiography study. Echocardiography 32: 135–143, 2015. 24661011 10.1111/echo.12581

[27] Bowman AW , Kovacs SJ : Assessment and consequences of the constant‐volume attribute of the four‐chambered heart. Am J Physiol Heart Circ Physiol 285: H2027–H2033, 2003. 12869381 10.1152/ajpheart.00249.2003

[28] Weyman AE , Wann S , Feigenbaum H , Dillon JC : Mechanism of abnormal septal motion in patients with right ventricular volume overload: a cross‐sectional echocardiographic study. Circulation 54: 179–186, 1976. 939018 10.1161/01.cir.54.2.179

[29] Walker RE , Moran AM , Gauvreau K , Colan SD : Evidence of adverse ventricular interdependence in patients with atrial septal defects. Am J Cardiol 93: 1374–1377, A1376, 2004. 15165917 10.1016/j.amjcard.2004.02.033

[30] Abd El Rahman MY , Hui W , Dsebissowa F , Schubert S , Gutberlet M , Hetzer R , Lange PE , Abdul‐Khaliq H : Quantitative analysis of paradoxical interventricular septal motion following corrective surgery of tetralogy of fallot. Pediatr Cardiol, 26: 379–384, 2005. 16374687 10.1007/s00246-004-0753-y

[31] Ostenfeld E , Stephensen SS , Steding‐Ehrenborg K , Heiberg E , Arheden H , Radegran G , Holm J , Carlsson M : Regional contribution to ventricular stroke volume is affected on the left side, but not on the right in patients with pulmonary hypertension. Int J Cardiovasc Imaging 32: 1243–1253, 2016. 27142431 10.1007/s10554-016-0898-9

[32] Smith J , Goetze JP , Sondergaard L , Kjaergaard J , Iversen KK , Vejlstrup NG , Hassager C , Andersen CB : Myocardial hypertrophy after pulmonary regurgitation and valve implantation in pigs. Int J Cardiol 159: 29–33, 2012. 21411159 10.1016/j.ijcard.2011.02.022

[33] Munkhammar P , Carlsson M , Arheden H , Pesonen E : Restrictive right ventricular physiology after tetralogy of Fallot repair is associated with fibrosis of the right ventricular outflow tract visualized on cardiac magnetic resonance imaging. Eur Heart J Cardiovasc Imaging 14: 978–985, 2013. 23364871 10.1093/ehjci/jet009

[34] Ersboell M , Vejlstrup N , Nilsson JC , Kjaergaard J , Norman W , Lange T , Taylor A , Bonhoeffer P , Sondergaard L : Percutaneous pulmonary valve replacement after different duration of free pulmonary regurgitation in a porcine model: effects on the right ventricle. Int J Cardiol 167: 2944–2951, 2013. 22995417 10.1016/j.ijcard.2012.08.012

[35] Heiberg E , Sjogren J , Ugander M , Carlsson M , Engblom H , Arheden H : Design and validation of Segment–freely available software for cardiovascular image analysis. BMC Med Imaging 10: 1, 2010. 20064248 10.1186/1471-2342-10-1 PMC2822815

